# The nutritional and feeding status of children living in selected residential child care facilities in Zambia: implications for programs and policies

**DOI:** 10.3389/fpubh.2024.1331907

**Published:** 2024-09-04

**Authors:** Zeina Makhoul, Mulemba Ndonji, Julie M. Long, Carolyn Moore, Edgar Lunda, Watson Mwandileya, Douglas Taren

**Affiliations:** ^1^SPOON, Portland, OR, United States; ^2^Access to Health Zambia, Lusaka, Zambia; ^3^Section of Nutrition, Department of Pediatrics, University of Colorado School of Medicine, Aurora, CO, United States

**Keywords:** undernutrition, residential care, care reform, Zambia, feeding, disability, orphanages

## Abstract

**Introduction:**

This study aimed to estimate the prevalence of undernutrition and risk of feeding difficulties and describe common feeding practices for children from birth to 10 years of age living in residential care in Zambia.

**Methods:**

This was a secondary analysis of de-identified cross-sectional data on 397 children living in 22 residential care facilities in four provinces. Child demographics, anthropometrics, hemoglobin levels, risk for feeding difficulties, and facility-level feeding practices were collected by a trained study team using *Count Me In*, a digital health app. Interviews with staff were conducted at 15 residential care facilities.

**Results:**

Around half of the study sample were boys (53.4%) and <5 years old (55.4%). Special healthcare needs were reported in 10.3% of the children, with cerebral palsy being the most common (3.5%). Underweight, stunting, wasting (using weight-for-length/height), and anemia were found in 22.4, 28.0, 7.1 and 54.7% of children, respectively, with higher rates in children with special healthcare needs and children <24 months old. Duration of residential care was positively associated with length/height-for-age but not weight-for-age or weight-for-length/height *z*-scores. A risk for feeding difficulties was found in 41.4 and 26.0% of children with and without special healthcare needs, respectively. Suboptimal bottle-feeding practices, including the use of altered nipples and poor caregiver-infant interactions, were observed for infants <12 months old. Residential care staff reported suboptimal diets in their facilities and gaps in knowledge and resources to meet children’s nutritional needs.

**Conclusion:**

These results demonstrate that a large proportion of children living in residential care in Zambia are at high risk for undernutrition and feeding difficulties and contribute to the small body of literature on children living in residential care, both in Zambia and globally. In the context of Zambia’s efforts to improve child nutrition and reform its alternative care, these findings can inform programming and policies for children living in residential care to fulfill their rights to health and family care.

## 1 Introduction

Globally, an estimated 105 children per 100,000 lived in residential care institutions in 2022 (1). This figure is likely an underestimate due to a lack of data systems, particularly in low- and middle-income countries, that can generate accurate and reliable estimates on this population of children. Residential care is defined in the UN Guidelines for the Alternative Care of Children as: “care provided in any non-family-based group setting, such as places of safety for emergency care, transit centers in emergency situations, and all other short- and long-term residential care facilities, including group homes (2).”

It is widely recognized that living in residential care has adverse impacts on children’s growth, development, and well-being, both during childhood and later in life (3, 4). The UN Guidelines on the Alternative Care of Children (2) affirm that children in alternative care should have access to adequate amounts of wholesome and nutritious food and appropriate supplementation, and their individual nutritional needs cared for. However, institutionalization exposes children to significant risks of undernutrition as well as multiple types of adversity including abuse, neglect, isolation, lack of stimulation, and toxic stress (4, 5). This exposure has a profound effect on children’s physical growth (6–8), nutritional status (6), cognitive development (6, 9), and socioemotional health, with exposure during early childhood being especially damaging (4).

These adverse effects are exacerbated in children with disabilities, who are at high risk for abuse and neglect in institutions (10) and have a baseline risk for undernutrition and developmental delays (11). Children with disabilities are more likely to be placed into residential care due to the lack of community-based healthcare, support services, stigma, discrimination, and poverty (4, 10, 12). Children with developmental disabilities, like cerebral palsy and Down syndrome, are particularly vulnerable to the harmful impacts of institutional settings and care practices (13). Additionally, many children with cerebral palsy, the most common childhood disability, are at increased risk for feeding difficulties and undernutrition due oral-motor impairments with subsequent swallowing difficulties and limited food intake (14–16).

In Zambia, an estimated 190 residential care facilities exist (17), and a reported 6,517 children under 18 years of age lived in residential care facilities in 2021 (18). Zambia has ratified the UN Conventions on the Rights of the Child (19) and the Rights of Persons with Disabilities (20), which affirm the rights of children, including children with disabilities, to health, family care, and community living. Zambia began care reform efforts to move away from residential care toward family care in 2001 under the leadership of the Ministry of Community Development and Social Services. Zambia has developed several key policy frameworks and guidelines (21) that prioritize family care and reintegration of children from residential care to communities and provide standards for alternative care, including: the Minimum Standards of Care for Child Care Facilities of 2014 (22), the National Child Policy of 2015 (23), the Alternative Care and Reintegration Guidelines of 2017 (24), and the National Framework for the Care of Children in Need of Care of 2019 (25).

In 2017, a nationwide assessment of residential care facilities in Zambia reported that many facilities did not meet the minimum standards of care in several areas, including nutrition, and found concerns around menu planning, diet diversity, and using food as a form of punishment (17). Two-thirds of children living in residential care were admitted by their parents or guardians (21), and common reasons were poverty, food insecurity, a disability or chronic illness (17, 26). Therefore, many children entering residential care in Zambia are already exposed to factors that increase their risk for undernutrition.

The 2018 Zambia Demographic Health Survey (DHS) (27) indicated high rates of undernutrition in the general pediatric population, with 34.6% of all children under 5 years old being stunted, 4.2% wasted, and 11.8% underweight (27). While child nutrition overall is a high priority in Zambia’s national development strategy, little is known about the nutritional status of children living in residential care. The sampling frames for the 2018 Zambia DHS and the currently ongoing 2023–2024 Zambia DHS (28) were restricted to households sourced by the national Census of Population and Housing (29) and thus, included children in home-based care only. Other national nutrition surveys (30) have also systematically excluded children living in residential care, contributing to data gaps for this population.

This study aimed to describe the nutritional and feeding status among children living in residential care in Zambia. Specifically, the objectives were to estimate the prevalence of undernutrition and feeding difficulties and explore common feeding practices among children from birth to 10 years of age living in 22 residential care facilities; understand nutrition and feeding-related support needed by children living in residential care; and identify the gaps and opportunities in nutrition and feeding services and policies for children living in residential care. This study was necessary to provide new knowledge that strengthens the evidence base and informs nutrition policies and programming for children living in residential care. It also aligns with the 2030 Sustainable Development ‘leave no one behind’ Agenda (31) by contributing often neglected data in support of Sustainable Development Goal (SDG) Target 2.2 to “end all forms of malnutrition, including achieving targets on stunting and wasting in children under 5 years of age” (32).

## 2 Materials and methods

### 2.1 Study design and period

We performed a secondary analysis of de-identified cross-sectional data collected at baseline between June 2017 and August 2021 through the *Improving Feeding and Nutrition Program* implemented in Zambia by SPOON and Access to Health Zambia (formerly called Catholic Medical Mission Board Zambia), and with permission from the Ministry of Community Development and Social Services, which is mandated to provide child protection services and oversight of residential care facilities. This program began in 2017 with the goal to improve feeding and nutrition outcomes for children with disabilities and children living in residential care – two groups of children highly vulnerable to undernutrition and often excluded from mainstream nutrition programs and policies. In this study, we present findings for three separate groups living at residential care facilities: children with no special healthcare needs, children with reported cerebral palsy, and children with reported special healthcare needs other than cerebral palsy.

#### 2.1.1 Ethical considerations

Research approvals for this secondary analysis were granted from the University of Zambia’s Humanities and Social Science Research Ethics Committee (Lusaka, Zambia), the Institutional Review Boards at St. Catherine’s University (St. Paul, Minnesota, USA), and the Colorado Multiple Institutional Review Board at the University of Colorado (Aurora, Colorado, USA). This was a retrospective study involving the analysis of de-identified data previously collected and therefore, no written informed consent was required. Verbal consent was obtained from interview participants and was deemed sufficient by the study’s ethics review committees. The study was conducted in accordance with the Declaration of Helsinki (33).

#### 2.1.2 Study settings and subjects

The sample included a purposive sample of 397 children from birth to 10 years old living in 22 residential care facilities across four provinces: Lusaka (*n* = 6), Southern (*n* = 6), Central (*n* = 6) and Copperbelt (*n* = 4). These provinces were selected because they had the highest number of children from birth to 10 years old living in residential care (17) and are conveniently in close proximity to Lusaka. Residential care facilities were included in the study if they were registered with the Ministry of Community Development and Social Services, served children from birth to 10 years old, and provided care for children for at least 9 months out of the year. No specific eligibility criteria were made for the size of the facility or whether or not they served children with disabilities. After permissions were obtained from the eligible facilities, all children birth to 10 years old living at the facilities, regardless of their disability status, were included in the study. All participating residential care facilities were privately run, as is common in Zambia. The capacity of the facilities ranged from 15 to 196 children. The number of facility staff varied widely and generally included administration (i.e., director or Catholic Sister-in-charge) and caregiving staff (i.e., caregivers) responsible for feeding children and other daily caregiving activities (e.g., bathing and dressing); and in some cases, a resident (or visiting) healthcare provider (e.g., a nurse, a medical officer, a physiotherapist), and a social worker.

### 2.2 Data collection

#### 2.2.1 Quantitative data collection

Child demographics, anthropometric measurements, anemia status, and feeding data were collected by a trained Zambia-based study team (a nutritionist, a physiotherapist, and a social worker) using *Count Me In*, a digital health data collection app designed by SPOON (34). A profile was created in *Count Me In* for each of the participating facilities. The study team members were set up with individual, secure *Count Me In* accounts and specific access permissions (for example, the study team had access to all residential care facilities while facility staff had only access to their site).

##### 2.2.1.1 Demographics

Child demographics included sex, date of birth and whether it was actual or estimated; date of admission to the residential care facility, and special healthcare needs diagnosis. For children without official documents, the child’s age was estimated by a healthcare staff based on the developmental milestones the child had reached at time of admission. In our sample, 108 out of 397 children had estimated dates of birth. Special healthcare needs were reported by the facility administration or caregiving staff; and were not a formal diagnosis by a medical doctor. *Count Me In* provided a list of conditions associated with increased risk for nutrition and feeding difficulties to select from: autism spectrum disorder, cerebral palsy, cleft lip/palate, cognitive impairment, Down syndrome, HIV/AIDS, heart disease/defect, hydrocephalus, seizure disorder/epilepsy, visual impairment, low birth weight (<2.5 kg), premature birth, and a category for other.

##### 2.2.1.2 Anthropometric measurements

Weight, recumbent length for children <24 months old, height for children ≥24 months old, and mid-upper arm circumference (MUAC) were measured using calibrated growth equipment and standardized measuring techniques (35) and entered into *Count Me In*. To determine if length and height were appropriate to measure, the study team first answered two Yes/No questions in *Count Me In*: “Is the child able to stand without assistance?” and “Is the child able to fully straighten legs?”. If “No” was selected to one or both questions, *Count Me In* concealed the length/height data entry field and revealed only the weight and MUAC data entry fields to the study team. Therefore, length and height measurements that could not be completed safely or accurately due to certain conditions (e.g., contractures) were not conducted (*n* = 2). The app generated *z-*scores for length/height-for-age, weight-for-length/height, weight-for-age, MUAC-for-age or MUAC cut-off points, based on the World Health Organization (WHO) growth standards (36). Children with poor growth were referred to the nearest health facility for further evaluation.

##### 2.2.1.3 Anemia screening

Anemia screening was conducted by measuring hemoglobin in capillary blood using the Hemocue Hb201+ analyzer (37). The heel for children <12 months old or finger for children ≥12 months old was pricked using a sterile safety lancet with a retractable needle. After wiping the first 1–2 blood drops with clean gauze, a 10 μL blood drop was collected in a Hemocue Hb201+ microcuvette. The filled microcuvette was then inserted in the Hemocue analyzer, which displayed hemoglobin values in g/dL. Hemoglobin values were entered in *Count Me In* and WHO recommendations on hemoglobin cut-off points for children (38) were used to identify the presence of anemia. *Count Me In* captured the presence of infection (e.g., malaria, fever) because hemoglobin concentrations are altered by the acute phase response to infection (39). No infections were reported in the study sample. The study team counseled facilities on iron-rich foods, hygiene, and sanitation; and on referring children with anemia to the nearest health facility for subsequent treatment.

##### 2.2.1.4 Mealtime screening

*Count Me In* guided the study team through screening children for risk for feeding difficulties by asking caregivers tasked with feeding about five mealtime domains (40–44): *Assistance* (support needed to eat or drink; options included full, some, or none), *Tools* (utensils used to eat or feed; options included bottle, spoon, cup, and/or fingers), *Texture* (food consistency the child consumed; options included formula milk, thin liquid other than formula, puree, mashed, soft and bite-sized, and/or regular foods), *Duration* (how long mealtimes lasted; options included < v10 minutes, 10–30 min, or >30 min), and *Frequent coughing/choking* (yes or no). *Count Me In* compared answers provided to what was developmentally expected at the child’s age at the time of the screening.

Answers that fell outside of the following parameters indicated a risk for feeding difficulties: for ages 0–6 months – received full assistance, fed with bottles only, and fed infant formula milk exclusively; for ages 6–12 months – received full or some assistance, fed with bottles, spoons, cups, and/or fingers, and fed infant formula, other thin liquids, purees, mashed, and soft and bite-sized foods; for ages ≥12 months – received some or no assistance, fed with spoons, cups, or fingers, and fed regular foods; and for all ages – completed mealtime in 10–30 min and did not frequently cough or choke. Children with cerebral palsy or Down syndrome were automatically considered at risk for feeding difficulties given the increased association of these disabilities with impaired feeding skills (13). For children with these conditions, *Count Me In* collected additional information on tools, food textures, and frequent coughing/choking.

##### 2.2.1.5 Facility-level mealtime best practices assessment

A facility-level assessment of feeding best practices involved observing a mealtime at the facility that included bottle feeding (<12 months old) and/or spoon feeding (≥12 months old); and answering questions in *Count Me In* based on these observations. If a mealtime observation was not possible at a facility, the study team asked caregivers or administrative staff instead. The questions aimed to understand how many caregivers (*all*, *most*, *half*, *a few*, or *none*) were following feeding best practices that promote safety, efficiency, skill-building and social development for infants and young children (45–48). Bottle-feeding practices of interest included following infant cues to stop/pause feeding, interacting with infants during feeding, offering infants breaks to burp, holding infants with head supported in arms, and using intact nipples. Spoon-feeding practices of interest included following child cues to properly pace feeding, interacting with children during mealtime, sitting at eye level with children during feeding, and children sitting together during mealtime and using small spoons appropriate for their age. The study team counseled facilities on any bottle- or spoon-feeding best practices that were not consistently followed by *all* caregivers.

##### 2.2.1.6 Data management

Deidentified data from *Count Me In* were transferred to STATA (version 17, StataCorp, College Station, TX) for statistical analyses. The data were checked for completeness and outliers. Extreme anthropometric *z*-scores and related growth measurements were excluded from the analysis (*n* = 10 length/height-for-age *z*-scores; *n* = 4 weight-for-age *z*-scores; and *n* = 2 weight-for-length/height *z*-scores) per WHO criteria (49). Distributions and frequencies for the raw data were then viewed. Dichotomous variables were created for each special healthcare need (e.g., reported cerebral palsy, 0 = not present, 1 = present). Children were grouped as having no special healthcare needs, having cerebral palsy, or having any other special healthcare need. The number of special needs that a child had was calculated by adding all the conditions together for each child. Descriptive data were tabulated based on the new groupings and presented as means and standard deviations for continuous variables and observations and percentage frequencies for categorical variables.

Undernutrition indicators, stunting, wasting, and underweight, were assessed using *z*-scores for length/height-for-age, weight-for-length/height, and weight-for-age, respectively. The severity of undernutrition was classified as normal, moderate, or severe according to the following *z*-score thresholds: normal (≥ −2), moderate (< −2 and ≥ −3), and (severe < −3) (36). In addition to weight-for-length/height *z*-scores, age-adjusted MUAC cut-offs were used to report levels of wasting as follows: ages 6–59 months: normal (≥12.5 cm), moderate (<12.5 cm and ≥11.5 cm), and severe (<11.5 cm); ages 60–120 months: normal (≥14.5 cm), moderate (<14.5 and ≥13.5 cm), and severe (<13.5 cm) (50). Anemia was measured using blood hemoglobin concentration and its severity was determined using cut-offs by age group as follows: 6–23 months: mild 9.5–10.4 g/dL, moderate 7.0–9.49 g/dL, and severe <7.0 g/dL; 24–59 months: mild 10.0–10.9 g/dL, moderate 7.0–9.9 g/dL, and severe <7.0 g/dL; and for 5–11 years: mild 11.0–11.4 g/dL, moderate 8.0–10.9 g/dL, and severe <8.0 g/dL (38).

##### 2.2.1.7 Statistical analysis

Our target sample size was 370 and was based on estimating an underweight prevalence of 32% with a 5% margin of error and a 95% confidence interval (based on *Count Me In* data for children living in residential care facilities in Zambia.) Statistical significance between groups was set at a *p*-value of <0.05. ANOVA was used to compare differences between the three special healthcare needs groups for continuous variables. Chi-squared tests were used to compare differences for categorical variables between children with no reported healthcare needs and children with healthcare needs other than cerebral palsy. We conducted multiple linear regression to identify associations between how long a child had received residential care prior to growth measurements (months of care) and anthropometric *z*-scores. Months of residential care was dichotomized into two categories: <24 months and ≥24 months. The regression models were adjusted for special healthcare needs groups (no special healthcare needs, other special healthcare needs, cerebral palsy), age at assessment groups (<24 months, 24–59 months, and ≥60 months), and sex.

#### 2.2.2 Qualitative data collection

We conducted semi-structured interviews with administrators (*n* = 8), social workers (*n* = 6) and a teacher (*n* = 1) at 15 residential care facilities to explore opinions on the causes of undernutrition among children living in residential care, care practices at residential care facilities, and gaps and opportunities for improving nutrition and feeding policies and programs for children living in residential care. Interviews were conducted in person at the facilities following a structured interview guide. Interview notes were captured in a notebook during the interview and entered into a spreadsheet immediately following the interview. Notes were entered into Microsoft Excel, with all responses organized by respondent and by question. Excel sheets were then created for each question and documented all responses to that question. One study team member read through responses for each question twice, identified preliminary themes, assigned each theme to a column within the Excel sheet, and sorted responses into the appropriate columns. Responses could be listed under more than one theme, and in those cases, we noted the portion of the response that aligned with the theme by using bold text. The same study team member then reviewed themes for completeness and accuracy and noted any questions or items for discussion. After the preliminary analysis, two members of the study team (one from SPOON and one from Access to Health Zambia) reviewed coding for all interviews, reconciled disagreements, and finalized coding and themes. Proportions were generated based on the number of responses listed under each theme.

## 3 Results

A total of 397 children living in residential care facilities (53.4% boys) were included in the study (Table 1). The average age of children was 54.0 ± 38.1 months old, ranging from 0.4 to 120 months old, with 55.4% of children younger than 5 years of age. Most of the children did not have a reported special healthcare need (89.7%). Of the special healthcare needs specified, cerebral palsy was most common (3.5% of all children) followed by HIV/AIDS (2.2%) and cognitive impairment (1.3%), and 5.7% of the children had “other” types of special healthcare needs reported (Table 1). Of the 41 children with special health needs, 60.9% had one condition reported and 31.7% children had more than one condition reported.

**Table 1:** Characteristics of children living in residential care facilities^1^.

	*n* = 397
Sex	
Female	185 (46.6%)
Male	212 (53.4%)
Age at assessment	
Mean age ± standard deviation (months)	54.0 ± 38.1
Age range (months)	0.4–119.9
Age at assessment groups	
<6 months	48 (12.1%)
6–11 months	34 (8.6%)
12–23 months	42 (10.5%)
24–59 months	96 (24.2%)
60–120 months	177 (44.6%)
Special healthcare needs present?	
Yes	41 (10.3%)
No	356 (89.7%)
Special healthcare needs^2^	
Cerebral palsy	14 (3.5%)
HIV/AIDS	9 (2.2%)
Cognitive impairment	5 (1.3%)
Low birth weight	2 (0.5%)
Seizure disorder/epilepsy	2 (0.5%)
Visual impairment	2 (0.5%)
Prematurity	2 (0.5%)
Autism spectrum disorder	1 (0.3%)
Down syndrome	1 (0.3%)
Other	23 (5.7%)
Number of special healthcare needs per child^3^	
One	25 (61.0%)
Two	13 (31.7%)
Three or more	3 (7.3%)

^1^Results presented are *n* (%), unless otherwise specified. ^2^Special healthcare needs are self-reported and not diagnosed by a medical doctor. The percentages shown are out of *n* = 397 for each special healthcare need. ^3^The percentages shown are out of *n* = 41 for number of special healthcare needs per child.

Overall, underweight, stunting, wasting (using weight-for-length/height), and anemia were found in 22.4, 28.0, 7.1 and 54.7% of children, respectively (Table 2). Only one child was identified with wasting based on MUAC. There were statistically significant differences for the prevalence of underweight (*p* < 0.001) and stunting (*p* < 0.01) between the three special healthcare needs groups, with children with cerebral palsy having the highest prevalence rates (69.2 and 71.4%, respectively) (Table 2). Children with no reported special healthcare needs had a significantly higher (*p* < 0.001) mean weight-for-age *z*-score (−0.83 ± 1.37) and length/height-for-age *z*-score (−1.02 ± 1.49) compared to children with cerebral palsy (weight-for-age *z*-score = −2.72 ± 1.29 and length/height-for-age *z*-score = −2.54 ± 1.93) and children with other healthcare needs (weight-for-age *z*-score = −1.52 ± 1.25 and length/height-for-age *z*-score = −2.21 ± 1.43). There were no differences between the three groups in weight-for-length/height *z*-scores or prevalence of wasting using weight-for-length/height. While hemoglobin concentration did not differ significantly between the three groups, the presence of anemia was significantly lower (*p* < 0.05) in children without special healthcare needs (52.0%) compared to children with cerebral palsy (60.0%) and children with other special healthcare needs (65.0%).

**Table 2:** Undernutrition indicators for children living in residential care facilities.^1^

	All groups *n* = 397	No special healthcare needs *n* = 356	Cerebral palsy *n* = 14	All other healthcare needs^2^ *n* = 27
Age at assessment (months)	54.0 ± 38.1	53.9 ± 38.3	72.7 ± 32.2	44.3 ± 35.3
Sex (female)	189 (47.1%)	165 (46.4%)	8 (57.1%)	12 (44.4%)
Weight-for-age *z*-score***	*n* = 393 −0.94 ± 1.40	*n* = 356 −0.83 ± 1.37	*n* = 13 −2.72 ± 1.29	*n* = 24 −1.52 ± 1.25
Prevalence of underweight^3,^***	*n* = 393	*n* = 356	*n* = 13	*n* = 24
Total	88 (22.4%)	71 (19.9%)	9 (69.2%)	32 (42.9%)
Severe	26 (6.6%)	19 (5.3%)	5 (38.5%)	2 (8.3%)
Moderate	62 (15.8%)	52 (14.6%)	4 (30.8%)	6 (25.0%)
Length/height-for-age *z*-score***	*n* = 378 −1.11 ± 1.53	*n* = 351 −1.02 ± 1.49	*n* = 7 −2.54 ± 1.93	*n* = 20 −2.21 ± 1.43
Prevalence of stunting^3,^**	*n* = 378	*n* = 351	*n* = 7	*n* = 20
Total	105 (28.0%)	90 (25.6%)	5 (71.4%)	10 (50.0%)
Severe	35 (9.3%)	29 (8.3%)	1 (14.3%)	5 (25.0%)
Moderate	70 (18.5%)	61 (17.4%)	4 (57.1%)	5 (25.0%)
Weight-for-length/height *z*-score (WL/HZ)	*n* = 211 −0.16 ± 1.35	*n* = 195 −0.14 ± 1.38	*n* = 3 −1.03 ± 0.39	*n* = 13 −0.37 ± 0.77
Prevalence of wasting based on WL/HZ^3^	*n* = 211	*n* = 195	*n* = 3	*n* = 13
Total	15 (7.1%)	15 (7.1%)	0	0
Severe	4 (1.9%)	4 (2.1%)	0	0
Moderate	11 (5.2%)	11 (5.6%)	0	0
Prevalence of wasting based on MUAC^3^	*n* = 220	*n* = 220	*n* = 0	*n* = 0
Total	1 (0.45%)	1 (0.45%)	0	0
Severe	0	0	0	0
Moderate	1 (0.45%)	1 (0.45%)	0	0
Hemoglobin (g/dL)	*n* = 180 10.7 ± 1.7	*n* = 144 10.8 ± 1.5	*n* = 10 10.9 ± 1.9	*n* = 26 10.1 ± 2.3
Prevalence of anemia^4,^*	*n* = 128	*n* = 98	*n* = 10	*n* = 20
Total	70 (54.7%)	51 (52%)	6 (60.0%)	13 (65.0%)
Severe	3 (2.3%)	1 (1.0%)	0	2 (10.0%)
Moderate	32 (25.0%)	22 (22.5%)	4 (40.0%)	6 (30.0%)
Mild	35 (27.3%)	28 (28.6%)	2 (20.0%)	5 (25.0%)

^1^Continuous variables are presented as mean ± standard deviation. Differences between groups analyzed using Chi-squared test for categorical variables and one-way ANOVA for continuous variables. *p < 0.05; **p < 0.01; ***p < 0.001. ^2^All other healthcare needs include autism spectrum disorder, cognitive impairment, down syndrome, premature birth, low birth weight, HIV/AIDS, visual impairment, seizure disorder/epilepsy, and other. ^3^Classification of underweight, stunting, and wasting was based on *z*-score thresholds: normal (≥ − 2), moderate (< −2 and ≥−3), and (severe < −3). ^4^Anemia was measured by blood hemoglobin concentration and its severity categorized using cut-offs by age group: 6–23 months: mild 9.5–10.4 g/dL, moderate 7.0–9.49 g/dL, and severe < 7.0 g/dL; 24–59 months: mild 10.0–10.9 g/dL, moderate 7.0–9.9 g/dL, and severe < 7.0 g/dL; and for 5–11 years: mild 11.0–11.4 g/dL, moderate 8.0–10.9 g/dL, and severe < 8.0 g/dL.

There were no significant differences between boys and girls in the prevalence of underweight, stunting, wasting (using weight-for-length/height), or anemia (Table 3). Underweight and stunting significantly differed by age at assessment (*p* < 0.001). Underweight was most prevalent in children younger than 6 months old (50.0%) while stunting was most prevalent in children 12–23 months old (63.4%). The presence of anemia was high in all age groups but with no statistically significant differences between groups (Table 3). Underweight (*p* < 0.05) and stunting (*p* < 0.001) were more prevalent among children who were living at residential care facilities for less than 24 months. There were no significant differences in the prevalence of undernutrition by age at admission (Table 4).

**Table 3:** Prevalence of underweight, stunting, wasting and anemia by age and sex for children living in residential care facilities.^1^

	Underweight^2^	Stunting^2^	Wasting^2,^^3^	Anemia^4^
Sex				
Female	43/184 (23.4%)	41/172 (23.8%)	8/97 (8.3%)	28/57 (49.1%)
Male	45/209 (21.5%)	64/206 (31.1%)	7/114 (6.1%)	42/71 (59.2%)
Age at assessment				
<6 months	23/46 (50.0%)**	22/45 (48.9%)**	3/45 (6.7%)	—
6–11 months	7/34 (20.6%)	8/34 (23.5%)	3/34 (8.8%)	
12–23 months	11/41 (26.8%)	25/40 (62.5%)	1/40 (2.5%)	13/23 (56.5%) 18/31 (58.1%)
24–59 months	17/95 (17.9%)	33/93 (35.5%)	8/92 (8.7%)	23/44 (52.3%)
60–120 months	30/177 (16.9%)	17/166 (10.2%)	––	16/30 (53.3%)
Months of care				
<24 months	36/98 (36.7%)*	51/91 (56.0%)*	5/77 (6.5%)	34/60 (56.7%)
≥24 months	5/39 (12.8%)	8/34 (23.5%)	1/17 (5.9%)	15/28 (53.6%)
Age at admission				
<12 months	19/56 (33.9%)	28/55 (50.9%)	4/53 (7.6%)	12/27 (44.4%)
≥12 months	34/103 (33.0%)	42/91 (46.2%)	5/61 (8.2%)	45/79 (57.0%)

Differences between groups analyzed using Chi-squared test for categorical variables. *p < 0.01; **p < 0.001. ^1^Results presented as n/N (%). N for each indicator varies due to missing data. ^2^Classification of underweight, stunting, and wasting was based on *z*-score thresholds: normal (≥ − 2), moderate (< −2 and ≥ −3), and (severe < −3). ^3^Wasting is based on weight for length/height and is only available for children 0–59 months old. ^4^Anemia was measured by blood hemoglobin concentration and its severity categorized using cut-offs by age group: 6–23 months: mild 9.5–10.4 g/dL, moderate 7.0–9.49 g/dL, and severe < 7.0 g/dL; 24–59 months: mild 10.0–10.9 g/dL, moderate 7.0–9.9 g/dL, and severe < 7.0 g/dL; and for 5–11 years: mild 11.0–11.4 g/dL, moderate 8.0–10.9 g/dL, and severe < 8.0 g/dL.

**Table 4:** Multiple linear regression analysis of anthropometric *z*-scores in children living in residential care facilities^1^.

	Weight-for-age *z*-scores *n* = 137			Length/height-for-age *z*-scores *n* = 125			Weight-for-length/height *z*-scores^2^ *n* = 94		
	ß-coefficient	SE	*p*-value	ß-coefficient	SE	*p*-value	ß-coefficient	SE	*p*-value
Months of care									
<24 months	ref			ref			ref		
≥24 months	0.354	0.254	0.166	0.684	0.296	**0.022**	−0.074	0.395	0.853
Special healthcare needs									
No special healthcare needs	ref			ref			ref		
Cerebral palsy	−2.275	0.381	**0.001**	−1.652	0.545	**0.003**	−1.206	0.899	0.183
All other healthcare needs	−0.583	0.281	**0.040**	−0.794	0.328	**0.017**	−0.201	0.414	0.629
Age at assessment									
<24 months	ref			ref			ref		
24–59 months	0.400	0.250	0.112	−0.026	0.277	0.924	0.326	0.292	0.267
≥60 months	0.796	0.297	**0.008**	0.909	0.339	**0.008**	--	--	--
Sex									
Female	ref			ref			ref		
Male	−0.147	0.202	0.469	−0.355	0.229	0.124	0.083	0.277	0.765

^1ß-coefficients for multiple regression for duration of care in months at residential care. Bold emphasis refers to variables with significant p-value. Ref, Reference value for each categorical variable identified in the table. 2Weight-for-length/height only includes children 0–59 months of age.^

Length/height-for-age *z*-score, modeled as a continuous variable, was significantly associated with duration of residential care, with months of care ≥24 months associated with a + 0.684 (*p* < 0.05) increase in length/height-for-age *z*-score compared to months of care <24 months (Table 4). There were no associations between duration of residential care and weight-for-age and weight-for-length/height *z*-scores. A cerebral palsy diagnosis had statistically significant, negative associations with weight-for-age *z*-scores (β = −0.275, *p* < 0.01) and length/height-for-age *z*-scores (β = −1.652, *p* < 0.01) (Table 4).

Mealtime screening identified a risk for feeding difficulties in 30.8% of the children. The use of age-appropriate feeding tools, food textures, and level of assistance; and appropriate mealtime duration were reported for the majority of children (96.1, 86.4, 89.2, and 84.7%, respectively) (Table 5). Frequent coughing and choking were reported for 2.3% of children. The use of age-inappropriate textures and frequent coughing and choking was most common in children with cerebral palsy compared to children with other special healthcare needs and children without special healthcare needs. More children with special heath care needs (other than cerebral palsy) were at risk for feeding difficulties (41.4%) compared to those without special healthcare needs (26.0%) but the difference was statistically non-significant.

**Table 5:** Screening for feeding difficulties among children living in residential care facilities.^1^

	All groups (*n* = 308)^2^	No special healthcare needs (*n* = 265)^3^	Cerebral palsy (*n* = 14)	All other healthcare needs^4^ (*n* = 29)
Age-appropriate feeding tool				
Yes	296 (96.1%)	254 (95.8%)	14 (100%)	28 (96.6%)
No	12 (3.9%)	11 (4.1%)	0	1 (3.4%)
Age-appropriate texture				
Yes	266 (86.4%)	237 (89.4%)	5 (35.7%)	24 (82.8%)
No	42 (13.6%)	28 (10.6%)	9 (64.3%)	5 (17.2%)
Age-appropriate level of assistance during mealtime^5^				
Yes	239 (89.2%)	212 (94.2%)	--	25 (86.2%)
No	29 (10.8%)	13 (5.8%)	--	4 (13.8%)
Appropriate mealtime duration^5^				
Yes	227 (84.7%)	199 (88.4%)	--	22 (75.9%)
No	41 (15.3%)	26 (11.6%)	--	7 (24.1%)
Frequently coughing and choking				
Yes	7 (2.3%)	1 (0.4%)	6 (42.9%)	0
No	301 (97.7%)	264 (99.6%)	8 (57.1%)	29 (100%)
At risk for feeding difficulties				
Yes	95 (30.8%)	69 (26.0%)	14 (100%)^6^	12 (41.4%)
No	213 (69.2%)	196 (74.0%)	0	17 (58.6%)

^1Differences between the No Special Healthcare Needs group and All Other Healthcare Needs group analyzed using Chi-square test for categorical variables. Cerebral Palsy group not included in analyses. 2N = 268 for level of assistance and mealtime duration due to missing data for 40 children. 3N = 225 for level of assistance and mealtime duration due to missing data for 40 children. 4All other healthcare needs include autism spectrum disorder, cognitive impairment, Down syndrome, visual impairment, seizures, and other. 5Questions skipped for children with cerebral palsy. 6Children with cerebral palsy are automatically considered to be at risk for feeding difficulties.^

**Figure 1:**
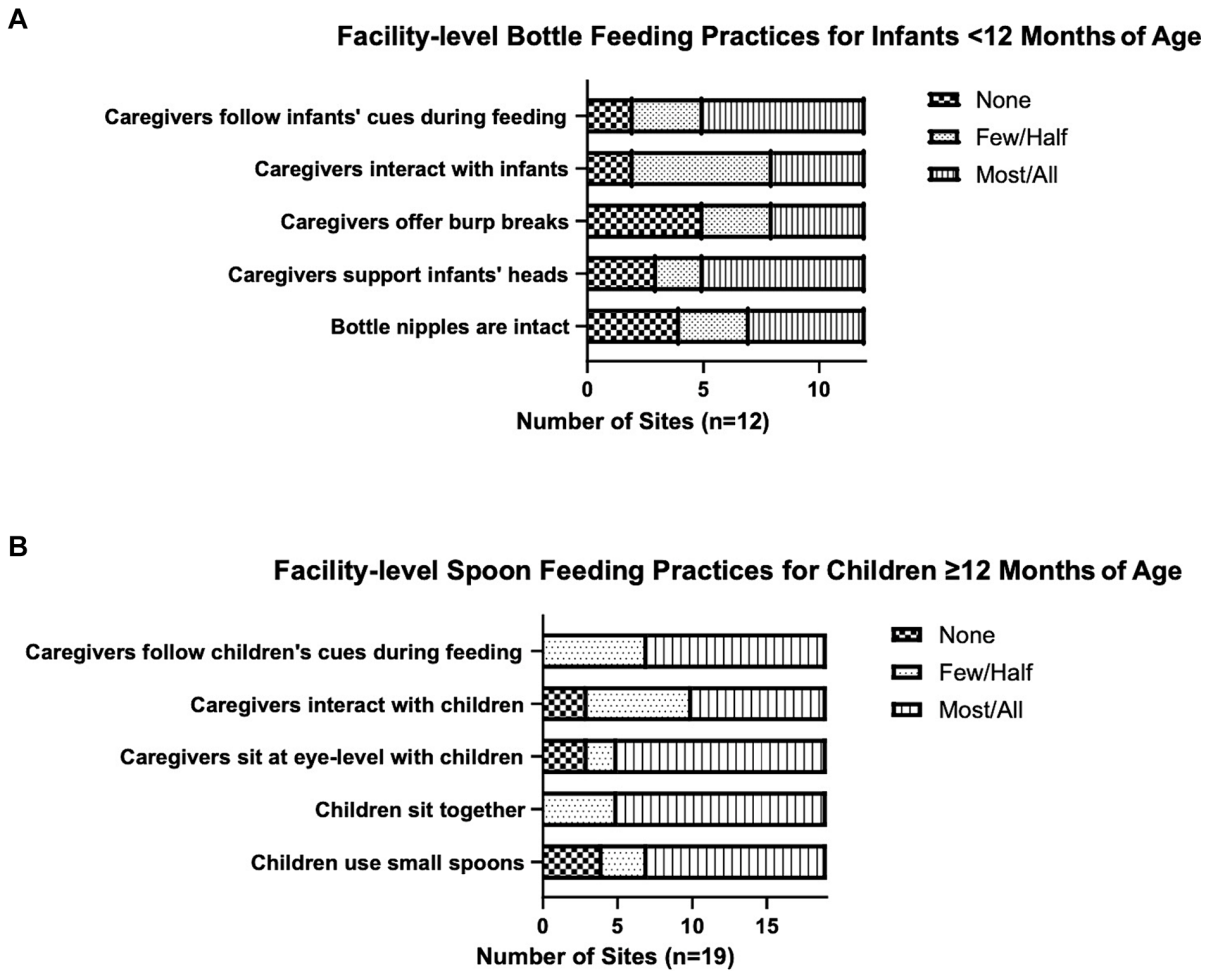
Feeding practices at residential care facilities. Twelve facilities answered questions around bottle feeding practices for children <12 months old **(A)** and 19 facilities answered questions around spoon feeding practices for children ≥12 months old **(B)**. None, few/half, and most/all refer to caregivers with three exceptions: the three categories refer to bottle nipples in “Bottle nipples are intact” and to children in “Children sit together” and “Children fed with small spoons”.

Bottle feeding for infants <12 months old and spoon feeding for children ≥12 months old were observed at 12 and 19 residential care facilities, respectively, with 10 facilities receiving both types of observations (Figure 1). When bottle feeding, *all* or *most* caregivers supported infants’ heads and followed infants’ hunger cues to stop feeding at more than half of the facilities (7/12). *All* or *most* infant bottle nipples were intact at less than half of the facilities (5/12). At a third of the facilities (4/12), *all* or *most* caregivers interacted with infants during bottle feeding or offered burp breaks (Figure 1A). At most facilities (14/19), *all* or *most* children ≥12 months old sat together for mealtime and *all* or *most* caregivers sat with them at eye-level. At more than half of the facilities (12/19), *all* or *most* children used appropriate size spoons and *all* or *most* caregivers followed children’s cues for feeding. Caregivers were observed interacting with children during mealtime at half of the facilities (9/19) (Figure 1B).

The residential care facilities interviewed housed an average of 46 children (range: 10–154). While all facilities cared for children 5 years and older, eight also accommodated children under 5 years old and five of these included infants under 12 months. All 15 facilities provided residential care services, with half (8/15) additionally serving children from the community. The facilities offered various services, with the most common being kitchen gardens (8/15), education (7/15), farming (6/15), healthcare (5/15), and reintegration (5/15). In interviews (Table 6), five residential care facilities noted challenges caring for children with special healthcare needs, and five reported that children at the facility had feeding difficulties. About half of the facilities (7/17) conducted regular growth monitoring; of these, half (4/7) took action based on results (diet modifications, increased monitoring, or referrals). Anemia testing was less common with only two facilities conducting regular screening, and six facilities screening when concerns arose. Among those that tested, just over half (5/8) acted on the results (supplementation, diet modifications, or referrals). All facilities served porridge and 10/15 reported enriching porridge with foods such as eggs, milk, sugar, or groundnuts. Facilities offered meat, poultry, fish, or eggs ranging from once a week (4/15) to five times a week (3/15). The majority of facilities offered at least two different types of fruits (11/15) and vegetables (11/15). Half of the facilities (8/15) reported that their staff received training on nutrition before or during employment. Facilities reported barriers related to food availability (4/15), cost (4/15), and knowledge in adequate nutrition and menu planning (4/15).

**Table 6:** Reported services, practices, and gaps at participating residential care facilities.

Theme		*n*/*N*	Comments
Clinical care practices			
Growth monitoring	Monthly	6/15	*Only 4 facilities reported taking action based on growth results, including making referrals or modifying the child’s diet.*
	Every 6 months	1/15	
	Infrequently or when concerned	4/15	
	None or unsure	4/15	
Anemia testing	Monthly	1/15	*Only 5 facilities reported taking action based on anemia results, including making referrals, giving iron supplements, or modifying the child’s diet.*
	Every 6 months	1/15	
	Infrequently or when concerned	6/15	
	None	7/15	
Nutrition supplementation (e.g., vitamin A, vitamin C, multivitamin, supplementary foods)	Any supplement	7/15	*2/15 facilities reported that supplements were provided when needed while others did not specify.*
	Multivitamin	4/15	
	Vitamin A	4/15	
	Vitamin D	2/15	
	Vitamin C	1/15	
	Iron	2/15	
	Deworming	2/15	
	None	8/15	
Nutrition and feeding practices			
Prolonged use of infant bottles (>18 months old)	Yes	2/6	*Bottle feeding was not a practice at the 9 facilities that served older children.*
	No	4/6	
Timely introduction of complementary foods	6–9 months of age	7/15	*6 facilities reported not having infants below the age of 12 months; 2 facilities reported introducing complementary feeding at 2 years of age.*
Introduction of cow’s milk	<12 months old	2/15	*7/15 facilities reported not using cow’s milk or not having a standard age for introduction.*
	1–3 years old	3/15	
	>3 years old	3/15	
Tea consumption	2–3 times/week	2/15	*Tea was commonly offered in the morning with breakfast.*
	Once/day	9/15	
	Twice/day	3/15	
	3 or more times/day	1/15	
Fruit and/or vegetable included in diet	Vitamin A-rich	8/15	*11 facilities reported including at least two types of fruits and two types of vegetables.*
	Vitamin C-rich	15/15	
Animal protein included in diet	1–2 times/week	6/15	*Examples included meat, fish, chicken, and eggs.*
	3–4 times/week	4/15	
	≥5 times/week	5/15	
Snacks included in diet	Only processed foods	6/15	*Nutrient-dense snacks included fruits and nuts.*
	Nutrient-dense foods	6/15	
	None or not often	3/15	
Staff training			
Training received	Any training	13/15	*Training included pre-service and in-service training for staff.*
	Childcare	7/15	
	Social work	7/15	
	Nutrition	8/15	
	None	2/15	
Gaps, challenges, and needs			
Risk factors for poor growth and health among children	Poor diet, feeding, and care practices	10/15	*8/15 facilities reported limited diet diversity and availability in communities served.*
	Illnesses or poor hygiene	6/15	
	Loss of parental care	2/15	
Barriers to providing a healthy diet at the facility	Insufficient resources	5/15	*One facility reported that consistent funding was the reason they did not have challenges.*
	High cost	4/15	
	Food availability	4/15	
	Poor nutrition knowledge	3/15	
	None	2/15	
Nutrition and feeding needs of the facility	Knowledge and information	5/15	*One facility reported that the increasing number of children were a challenge.*

## 4 Discussion

We examined the nutritional status and risk for feeding difficulties among children living in 22 residential care facilities in Zambia and explored common feeding practices in residential care. We found a high prevalence of undernutrition in this population with the risks being generally higher for younger children and children with special healthcare needs; and that one in three children were at risk for feeding difficulties. We also found that a longer duration in residential care was associated with a higher mean length/height-for-age *z*-score. We observed suboptimal bottle-feeding practices for infants younger than 12 months old, including the use of altered bottle nipples and poor caregiver-infant interactions during feeding.

### 4.1 Nutritional status of children living in residential care

This study found a high prevalence of stunting (28.0%), underweight (22.4%), wasting using weight for length/height (7.1%), and anemia (54.7%) among children from birth to 10 years of age living in residential care. For children under 5 years of age, prevalence estimates were 41.5, 26.8, 7.1 and 55.1%, respectively, and were generally higher compared to the 2018 Zambia DHS levels for stunting (34.6%), underweight (11.8%), wasting (4.2%), and anemia (58.0%) (27), which only included children in home-based care (29). It is uncertain if the inclusion of children living in residential care in the DHS would have significantly increased the national undernutrition prevalence rates in Zambia.

We found that, overall, children younger than 24 months were more likely to be undernourished, with stunting highest among children 12 to 23 months old at an elevated rate of 62.5% (*p* < 0.001). Our findings are in agreement with the age-disaggregated 2018 Zambia DHS rates for stunting, showing the prevalence peaking at 46% among children 18–23 months old (27). Our findings align with the global consensus on the importance of optimal nutrition and care during the first 1,000 days of life. Stunting results from chronic undernutrition, repeated infections, and inadequate psychosocial stimulation, among other environmental factors (51, 52). Stunting that occurs in early childhood, as seen in our study sample, has adverse functional consequences for the child, including frequent illnesses, poor cognition and learning capacity, and delayed development in other domains like language, sensory, and motor functions (53–55), with long-term economic and health risks (55, 56).

While literature shows that institutionalization of children is strongly linked to poor growth (4, 8, 57), recent data on undernutrition in residential care is scarce. Limited estimates from Africa align with our findings. A 2020 systematic review found varying rates of stunting (9–72%), wasting (0–27%), underweight (7–79%), and anemia (3–90%), with younger children faring worse (57). Four African studies (Ethiopia, Kenya, Malawi) from 1991 to 2014 were included in the review. A 2021 survey in Ghana reported 40% stunting, 22% underweight, and 15% wasting among children under 5 living in residential care (58). In Zambia, a 2020 study of 450 children living in residential care noted all children were below the WHO median for weight for age, with weight loss due to poor nutrition. The study did not include the prevalence of stunting, wasting, underweight, or anemia (59).

We found that children with cerebral palsy living in residential care were overall 3 times more likely to be stunted, 3.5 times more likely to be underweight, and 10 times more likely to be severely underweight compared to children without reported special healthcare needs. Undernutrition in children with cerebral palsy is highly prevalent in low- and middle-income countries (14–16, 60–66). Underweight was reported in 42%, and 65% of children with cerebral palsy in Uganda (66) and Ghana (65), respectively. A meta-analysis found that overall, 72–98% of children with cerebral palsy from Bangladesh, Indonesia, Nepal, and Ghana had at least one form of undernutrition (16). However, none of these studies were performed in residential care settings. Dangerous feeding practices like force feeding, inappropriate food textures, and unsafe child positioning during feeding, are common for children with disabilities in institutional settings (8, 13, 67). The high burden of undernutrition among children with cerebral palsy in this sample was likely due to several interlinked factors, some of which might be a direct result of their oral-motor impairments affecting chewing and swallowing and increased energy requirements, while others might be related to poor feeding, nutrition, and care practices, which can be difficult for caregivers, particularly in the absence of adequate training and resources (68).

Existing literature underscores the detrimental effects of prolonged residential care on children’s growth, health, and overall well-being (4, 7, 8). A 2020 meta-analysis highlighted that extended institutionalization is correlated with more pronounced growth delays (4). However, our cross-sectional analysis revealed a contrasting finding: a longer stay in residential care was associated with a higher length/height-for-age *z*-score after adjusting for special healthcare needs, age, and sex. This association was not observed for weight-for-age or weight-for-length/height *z*-scores. The inability to separate the duration of residential care from various child-level and residential care factors that could influence growth complicates the analysis (4). Additionally, it is likely that the children in our study experienced significant undernutrition before entering residential care, often stemming from the food-insecure environments they came from (17, 26). However, this conclusion could not be confirmed within the scope of our study. Moreover, it is important to note that the prevalence of stunting remained elevated for children with longer duration of care.

We did not investigate the risk factors for poor growth and anemia status in our sample and the timing of their exposure, whether before admission to residential care or while living in residential care. Our qualitative findings indicated that several residential care facilities had sub-optimal dietary practices, such as serving tea, which increases the risk for iron deficiency anemia (69, 70); and infrequently providing iron- and protein-rich foods, potentially leading to undernutrition. Similar sub-optimal practices were observed in a 2016 qualitative study of residential care facilities for adolescent girls in Uganda, where children’s right to food was not met in three out of five facilities, with many receiving only one meal per day (71). Many facilities in our study also struggled to offer diverse and adequate diets and lacked proper monitoring of children’s nutritional status. While our findings shed light on potential risk factors, further robust research is needed to explore causal pathways and solutions to undernutrition among children living in residential care in Zambia.

### 4.2 Feeding difficulties and feeding practices

Feeding difficulties refer to a wide range of delays or problems with eating or drinking, such as developing new feeding skills, maintaining a safe position for mealtime, chewing food, and safely swallowing (45). They are most common in children with developmental disabilities, with a global prevalence of 30–80%, but also exist in 25–45% of children without disabilities (72, 73). Lengthy meals, inability to graduate to advanced food textures, delay in self-feeding, prolonged use of infant bottles, and frequent coughing or choking are common indicators that a child might be at risk for feeding difficulties (45). In this cohort, all children with cerebral palsy were automatically classified as at risk for feeding difficulties due to the strong link between cerebral palsy and impaired feeding skills (13). We found that 41.4% of children with other special healthcare needs and 26.0% of children without special healthcare needs were at risk for feeding difficulties, which are comparable to the global pediatric prevalence estimates (73).

Feeding data on children living in residential care are rare. A recent retrospective analysis of programmatic data of 3,335 children birth to 18 years old in institutional care in six countries found that 5.9% of children without disabilities versus 29.7% of children with disabilities had a feeding difficulty (67). The higher prevalence in our study could be partially due to differences in how feeding difficulties were defined and identified. When interpreting findings related to feeding difficulties, it is important to bear in mind that feeding is a co-occupation involving the feeder (i.e., the caregiver) and the child. As a result, it can be difficult to separate the two when evaluating risk based on a screening. For example, delayed introduction of advanced food textures could indicate a poor practice by the feeder or a response to an actual chewing difficulty experienced by the child. Additionally, the mealtime screening used in this study only identified risk and did not diagnose feeding difficulties; therefore, it is likely that we identified some children who were at risk but not with actual feeding difficulties. Any suspected feeding difficulty should be investigated further through mealtime observations.

Feeding practices are important determinants of children’s nutrition, development, and health outcomes (74). Optimal feeding practices are responsive and support safety, skill-building, and social interactions (75). While some positive feeding practices were observed, none of the residential care facilities entirely followed best practices for bottle or spoon feeding. This was especially true for facilities that served infants, with altered bottle nipples observed at more than half of the facilities. Enlarging the hole in the tip of the bottle nipple is a common practice in institutions used to speed up feeding or feed pureed food from a bottle; and is a choking and contamination hazard (8). Additionally, consistent caregiver-infant interactions during feeding were only observed at a third of the facilities. By their nature, institutional settings do not allow for close caregiver-infant interactions. Feeding offers daily opportunities for caregivers to provide stimulation to infants through interactions, minimizing some of the adverse effects that institutionalization has on infants (76, 77). Other common practices included not following the infant’s cues for hunger and fullness when bottle feeding; and inappropriate pacing when spoon feeding. Similar practices have been noted by the very few studies that examined feeding practices in institutions (6, 8, 67).

### 4.3 Limitations

The study had several limitations. First, because this was a secondary analysis on programmatic data and not originally intended to study the nutritional status of children living in residential care, we could not establish the determinants of undernutrition in this population. Undernutrition among children living in residential care can stem from various factors beyond just the quality of care provided by the facility. We acknowledge the unavailability of data on children prior to their admission to residential care, which restricts our ability to fully evaluate the impact of institutionalization on their nutritional status. This also limits our understanding of how residential care compares to the home environments the children came from. The challenge lies in disentangling the duration of care from various child-level and residential care factors that could potentially influence growth. Consequently, the finding regarding the positive association of duration of care with anthropometric *z*-scores should be interpreted cautiously. Second, the analysis was conducted on a purposive sample from residential care facilities located only in Lusaka, Central, Copperbelt, and Southern provinces, limiting the generalizability of the results to the larger population of children living in residential care in Zambia. Third, age was estimated for 108 children based on developmental milestones achieved rather than more reliable methods like bone and dental evaluations (78). The interpretation of many of the study indicators relied on the child’s age and, therefore, should be interpreted with caution. Fourth, special healthcare needs were reported by caregivers, leaving us unable to confirm diagnoses. It is possible that more children had special healthcare needs than indicated. Finally, errors in growth measurements and their interpretations for children with cerebral palsy could have occurred. Length or height are challenging to measure for children with cerebral palsy, particularly children with contractures (79, 80). While we took measures to minimize inaccurate length and height data by not conducting these measurements on children who are unable to stand independently and straighten their legs, errors could still have occurred. Also, children with cerebral palsy often grow differently than children without cerebral palsy; therefore, comparing their growth to the WHO growth standards may overestimate their prevalence of undernutrition (80). We had to rely on the WHO growth charts since cerebral palsy-specific growth charts appropriate for low- and middle-income countries do not exist. Still, given the paucity of nutrition data on children with cerebral palsy living in residential care, it was important to include them in the analysis for descriptive purposes.

### 4.4 Implications for policy and programs

Zambia has set national goals to reduce childhood stunting and wasting. While the country has made progress toward these goals, with national stunting rates decreasing from 52.5% in 2002 to 34.6% in 2018, and wasting from 6.2 to 4.2% among children under 5 years old (81), and Zambia’s National Food and Nutrition Policy recognizes orphans and vulnerable children as a nutritionally vulnerable group (82), it appears likely that this progress has not reached children living in residential care. This study demonstrates high rates of undernutrition in children living in residential care, particularly young children and children with special healthcare needs, and therefore, validates the need to include these children in efforts to achieve Zambia’s nutrition goals.

Zambia also prioritizes family care and family reintegration in its care reform efforts, including training, policy development, and family support pilots (21). These efforts should consider the high risk for undernutrition and feeding difficulties among children living in residential care and have appropriate processes and services in place to support the child and parent/caregiver before, during, and after reintegration. For example, children should be screened for growth delays and nutritional deficiencies, and children with disabilities for feeding difficulties before, at the time of, and after reintegration; and services that support families should be provided, including nutrition and feeding support, to ensure stable reintegration (5). As Zambia transitions away from a residential care system, it is important to ensure that children’s nutritional and care needs are met to prepare children and families for a successful reintegration to family care (5). This can be achieved by improving training for residential care staff in child development, nutrition, feeding, and disability.

The *Improving Feeding and Nutrition Program* that generated data for this study is one example of a program aimed to improve nutrition and feeding practices for children living in residential care and children with disabilities in Zambia. Access to Health Zambia and SPOON have trained master trainers from the Ministry of Community Development and Social Services and the Ministry of Health, who in turn trained key residential facility staff and caregivers in nutrition and feeding best practices; supported the use of *Count Me In* at residential and community sites; and advocated for inclusive nutrition policies, programs, and investments that respond to the needs of children living in residential care, children at risk of family separation, and children with disabilities. Our program experience has demonstrated the feasibility of training and mentoring to improve nutrition and feeding practices in family strengthening and residential care settings; the importance of a digital tool to facilitate nutrition care delivery while generating data; and the critical role of data to support government stakeholders in filling gaps in nutrition policies and services (83).

### 4.5 Further research and recommendations

Our main recommendations from this study are:

- Increase the availability of nutrition and feeding data on children living in residential care through additional research studies, the inclusion of children living in residential care in Zambia’s routine nutrition surveillance (with data disaggregated by type of care and disability status), or the use of existing tools like the UNICEF’s Protocol for Data Collection in Residential Care (84). We recommend data collection on stunting, wasting, underweight, anemia, feeding difficulties, and facility-level feeding practices, to improve comparability and to identify trends over time.
- Prioritize children living in residential care and children at risk of separation from their family as high-risk groups for undernutrition in nutrition policies and strategies; and assess and improve their access to appropriate nutrition and feeding services, including for young children and children with disabilities.
- Ensure nutrition and feeding practices are represented in the implementation of Zambia’s child care reform strategy. This should include: supporting families at risk of separation or in the reintegration process to meet children’s nutrition and feeding needs; strengthening skills of service providers in alternative care and nutrition to support families and meet the nutrition needs of children living in residential care, including children with disabilities; increasing awareness of the potential adverse effects of institutionalization on children’s growth and development; and, given the established risks associated with institutionalization, accelerating efforts to support family care for all children in Zambia.

## 5 Conclusion

This study identified high rates of undernutrition and risk of feeding difficulties among children birth to 10 years old living in residential care in Zambia. Among this population, children under 2 years old and children with special healthcare needs, particularly children with cerebral palsy, were at an increased risk. Despite its limitations, the study contributed to filling a gap in nutrition data in Zambia, and demonstrated that existing national estimates of undernutrition do not necessarily capture the needs of children living in residential care. The study also demonstrated the need to include children living outside of family care in routine data collection efforts to “count *all* children” and more accurately assess progress toward SDG Target 2.2. Our findings can be used to recommend increased attention to this population of children in the context of Zambia’s nutrition and care reform agendas and to support program decisions and key stakeholders in their efforts to improve child nutrition and care reform systems to fulfill children’s rights to health and family care.

## Data Availability

The raw data supporting the conclusions of this article will be made available by the authors, without undue reservation.
